# Depicting patient-reported outcome measures within directed acyclic graphs: practice and implications for causal reasoning

**DOI:** 10.1007/s11136-025-04007-9

**Published:** 2025-06-27

**Authors:** Matthew Franklin, Tessa Peasgood, Peter W. G. Tennant

**Affiliations:** 1 Sheffield Centre for Health and Related Research (SCHARR), School of Medicine and Population Health, The University of Sheffield, Regent Court, 30 Regent Street, Sheffield, S1 4DA UK; 2 Leeds Institute for Data Analytics, Level 11 Worsley Building, University of Leeds, Leeds, LS2 9NL UK; 3 School of Medicine, University of Leeds, Worsley Building, Leeds, LS2 9NL UK; 4 Alan Turing Institute, British Library, 96 Euston Road, London, NW1 2DB UK

**Keywords:** Outcome, Measure, Formative, Reflective, Causal, DAG

## Abstract

**Purpose:**

Estimating causal effects of an exposure (e.g., health condition or treatment) on a patient-reported outcome measure (PROM) can have complications depending on the relationship between the PROM’s indicators and construct(s). Using directed acyclic graphs (DAGs) as visual tools, we show how to represent a PROM’s potential internal causal relationship between its indicators and latent construct(s), then explain the implications when also accounting for external variables when estimating causal effects within observational data.

**Methods:**

Measurement theory suggests a PROM’s relationships between its items/indicators and latent construct(s) is reflective (construct causes the indicators) or formative (indicators cause the construct). We present DAGs under reflective and formative model assumptions when the PROM is unidimensional (e.g., Patient Health Questionnaire-9 [PHQ-9] representing depression severity) or multidimensional (e.g., EQ-5D representing health-related quality-of-life).

**Results:**

Unidimensional PROMs under a reflective model can be analysed like other unidimensional outcomes (e.g., mortality) to estimate causal effects, thus don’t require additional consideration. In comparison, each indicator of a multidimensional construct under a formative model needs specific consideration to ensure relevant external variables are appropriately conditioned to estimate causal effects.

**Conclusion:**

Multidimensional outcome constructs formed under a formative model increases the complexity of causal analyses. Despite this, multidimensional measures may particularly aid with a variety of ‘outcome-wide’ studies when assessing exposures that may be beneficial for some outcomes but harmful for others. Thus, we have taken important steps to supporting such studies in observational settings by showing how PROMs can be incorporated into DAGs to inform such causal analyses.

## Introduction

Health-related outcome measures are commonly used to assess the comparative effectiveness of interventions, explore the determinants of population health, monitor the performance of healthcare providers, and guide clinical decision-making at individual and group levels [1–5]. Outcome measures may be completed by patients, i.e., patient-reported outcome measures (PROMs), by a designated expert (e.g., clinician), or a designated proxy (e.g., carer or loved one) [6]. The design of such measures depends on the latent concept(s) that the instrument is trying to measure, i.e., the intended ‘construct’ of the measure. As such, constructs are the postulated attributes that an investigator hopes to capture with an outcome measure [7]. There is an assumed relationship between the indicators of a measure (e.g., the question items) and the measure’s construct(s), although many indicators may load onto (i.e., be grouped to represent) more than one construct [8, 9].

Dimensionality refers to the number of constructs that a measure represents: unidimensional measures represent a single construct, whereas multidimensional measures represent two or more constructs. Some outcome measures are designed to be unidimensional; for example, the Patient Health Questionnaire-9 (PHQ-9) [10, 11]. Although the PHQ-9 contains nine items, each item is intended to measure the single (unidimensional) construct of depression severity. In contrast, some outcome measures are purposely designed to be multidimensional, such as the EQ instruments, whereby the EQ-5D represents five health dimensions within a single measure: mobility, self-care, usual activities, pain/discomfort, anxiety/depression [2, 12]. The EQ-5D is purposely multidimensional, with each EQ-5D item representing its own health dimension, but it is also suggested that the EQ-5D represents the single higher-level construct of health-related quality-of-life (HRQoL) [2, 13].

According to measurement theory, the relationship between the items within an outcome measure (i.e., indicators) and the latent construct(s) measured by that measure may be either reflective or formative [8, 9]. In a reflective model, the construct causes the indicators and are hence sometimes called ‘effect indicators’, whereas in a formative model the indicators cause the construct and are hence sometimes called ‘causal indicators’ [8, 9]. The assumed relationship between the indicators and the latent construct has conceptual and practical implications. For example, in a reflective model, the latent construct is thought to exist regardless of how and whether we measure it. The indicators are hence conceptually interchangeable because it does not matter which specific items we use, beyond having enough information for accurate measurement. In comparison, formative indicators are not interchangeable because each indicator contributes a specific meaning to the construct: when one indicator is changed, this leads to a fundamental change in the construct under study. A subsequent implication is that to understand and interpret the external relationship between an outcome measure and any other variable, e.g., to explore determinants of health, hence requires a clear understanding of the measure’s internal relationships. Causal graphs can aid with this aspect.

Causal directed acyclic graphs (DAGs) are an increasingly popular visual tool for representing the hypothesised causal relationships between variables. DAGs are more broadly associated with Structural Causal Models (SCMs) as a framework for understanding and analysing causal relationships between variables, particularly when dealing with observational data, which have been popularised by experts in causal inference including Judea Pearl, James Robins, and Miguel Hernan [14–17]. In health research, DAGs are commonly used for identifying appropriate variables for conditioning when estimating causal effects in observational studies [18]. However, DAGs may also be used to consider a range of analytical concepts and issues. In the following, we introduce the practice and implications of depicting outcome measures within DAGs, particularly for outcome measure users who may be less familiar with causal inference and DAGs. We begin by briefly introducing causal inference in observational data as a distinct and important task, and DAGs as a useful tool for supporting this task. We then introduce why this is relevant for outcome measure development and use, then discuss how to operationalise reflective and formative measurement models within DAGs, and the implications for causal inference involving outcome measures. For descriptive purposes, our example outcome measures within this article are PROMs (i.e., the patient-reported PHQ-9 and EQ-5D); however, the logic is applicable to any sort of outcome measure and associated use when the interest is the causal effect of an exposure on an outcome (or outcomes) represented by an outcome measure that has a purported construct(s) and related indicators.

## Causal inference and its relevance to outcome measures: a brief overview

Determining the causal effect of an exposure (e.g., treatment or health condition) on a health outcome (e.g., mortality or quality of life) is a key aim of applied health research, and of value in any health or healthcare setting. In many of these research and practical settings, outcome measures will represent causal outcomes of interest. For example, researchers and health and policy practitioners may be interested in the effect of providing Cognitive Behavioural Therapy (CBT), rather than counselling, on depression severity as quantified by the PHQ-9. To estimate this effect, we could conduct a randomised controlled trial (RCT) of CBT versus counselling. However, RCTs are not an option when the exposure of interest cannot practically or ethically be experimentally assigned. For example, if we wanted to know the causal effect of having cancer, compared with not having cancer, on HRQoL, then this cannot be studied by experiment since we cannot practically or ethically assign people to having cancer or not. Consequently, we are often required to estimate causal effects in observational or quasi-experimental settings, where the exposure is not under direct control. This is notoriously challenging, but in recent years has become more formalised with an increasing recognition of causal inference as a distinct scientific task that requires distinct methods. With a focus on estimating counterfactual scenarios and hypothetical interventions, causal inference differs from description (i.e., summarising data features) and prediction (i.e., identifying patterns and forecasting), because it requires external knowledge of how the data came into being, known as the data generating process [19]. While this external knowledge may be identified and communicated in a variety of ways, DAGs have become a popular tool for this purpose because of their relative ease of use and transparency.

## Directed acyclic graphs (DAGs): overview and definitions

**Fig. 1:**
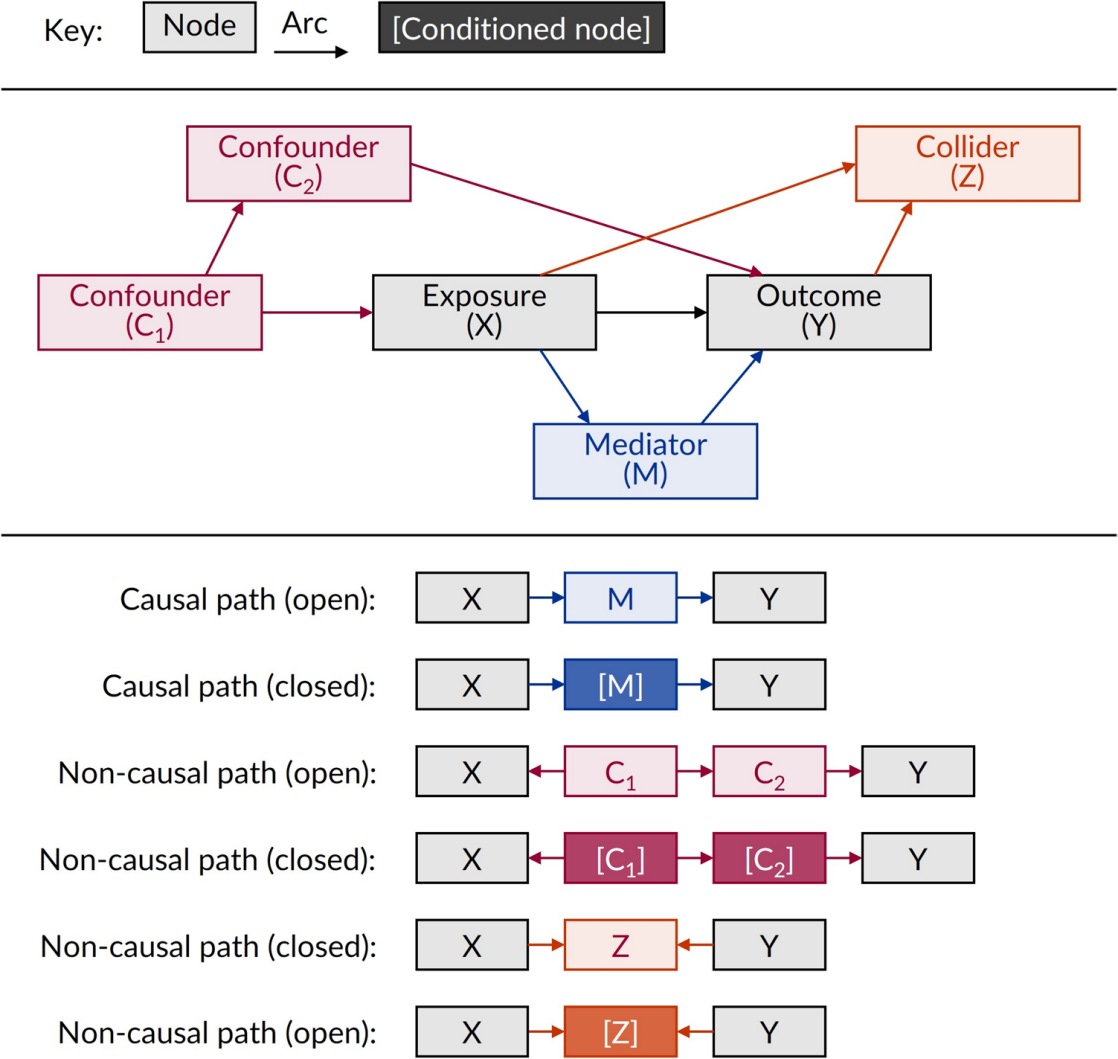
Simple illustration showing the main features of a directed acyclic graph. *Footnote*: variables within a directed acyclic graph (DAG) are represented by nodes. A unidirectional arrow (or ‘arc’) path signifies the first variable (i.e., parent node) is thought to cause the second (i.e., child node). Paths may be open (transmitting an association) or closed (not transmitting an association). Causal paths flow in the same direction, while non-causal paths do not. Because a DAG is acyclic, no single measure of a variable may cause itself. The average causal effect of a specified *exposure (X)* on a specified *outcome (Y)* is the combination of all causal paths between X and Y. In theory, this may be estimated from the conditional association between X and Y, if an appropriate set of variables are conditioned so all causal paths are open and all non-causal paths are closed. This requires conditioning on *confounders* (C_1_, C_2_), but not conditioning on *mediators* (M) or *colliders* (Z). Conditioning can be done using methods such as multivariable regression; conditioning in Figure is depicted using [Conditioned node], e.g., [C_1_] closes a non-causal path, whereas [M] closes a causal path and [Z] opens a non-causal path

Formally, DAGs are non-parametric diagrammatic representations of the hypothesised data-generating process for a set of variables (and measurements thereof) in a specified context [18]. More simply put, DAGs provide a simple and transparent way to identify and demonstrate current knowledge, theories, and assumptions about the causal relationships between variables [18]. The DAG structure can be used to identify which variables must be conditioned (e.g., adjusted for within a multivariable regression model) to estimate a particular causal effect of interest. Several introductory articles about the nature and use of DAGs already exist [18, 20, 21]. Although there are other graphical tools that can help researchers conceptualize and guide their research, such as structural equation modelling (SEM) diagrams, DAGs have become a key tool for studies that are explicitly interested in estimating causal effects [22]. As such, we briefly introduce some key DAG features and definitions provided by Tennant et al. [18], before focussing on some specific considerations relating to outcome measures as our unique contribution to the existing literature. Figure 1 and associated footnote provides a summary of key DAG features. Key DAG-related terms are also presented and described in Table 1, aspects of which have been influenced by other graphical tools such as SEM diagrams [22].

**Table 1:** Key DAG-related terms with visual representation and a brief description

Key term	Visual representation	Brief description
*Node*		Nodes represent variables (or measurements thereof) within a DAG
Individual variable		An individual variable represents a single measurement of a variable. Using SEM notation these variables are typically depicted as single-outlined rectangles
Composite variable		A composite variable is a single variable created by combining two or more individual variables. These variables are depicted as double-outlined rectangles, as recommended by Berrie et al. [32]
Latent variable		A latent variable is a variable that cannot be directly observed but can be inferred from other, directly observable variables. Using SEM notation these are typically depicted as ellipses
Conditioned variable		A conditioned variable is a variable that has been conditioned on, e.g. which has been restricted to a single value, stratified over or adjusted for, by including as a covariate within a regression model. A conditioned variable is often depicted by adding a box around a node, but this is not compatible with SEM notation where it is common to use darker shading. Thus, we depict conditioning both with darker shading and by adding square parenthesis around the name of the variable within the node
*Arrow (aka., arc)*		Nodes may be connected by a unidirectional arrow (or 'arc') to signify that the first variable is thought to cause the second, with the hypothesised direction of causality represented by the direction that the arrow is pointing. All arrows are unidirectional within a DAG (i.e., directed and acyclic), meaning that no measure of a variable can cause itself
Probabilistic		Probabilistic refers to a connection between variables where the outcome is not fully explained, meaning that knowledge of the value of all the causes of the outcome is not sufficient to know the value of the outcome with certainty. Depicted as a single lined arrow
Deterministic		Deterministic refers to a connection between variables where the outcome is fully explained, meaning that knowledge of the value of all the causes of the outcome is sufficient to know the value of the outcome with certainty. These arrows are depicted as double-lined arrows, as recommended by Berrie et al. [32]
*Paths*		Paths are connections between variables made up of one of more arrows
Causal path		Causal paths are paths where all arrows flow in the same direction. To estimate the average causal effect of an exposure on an outcome from an association using the back door criterion, all causal paths should be left open
Non-causal path		Non-causal paths are paths where the arrows do not all flow in the same direction; they include confounding paths (or back door paths) and collider paths. To estimate the average causal effect of an exposure on an outcome from an association using the back door criterion, all non-causal paths should be closed
*Kinship terminology*		The relationship between variables within a DAG is often described using kinship terminology. For a particular variable, all upstream causes are referred to as ancestors and all downstream consequences are referred to as descendants
Child		A direct descendent of a particular variable is known as a ‘child’ of that variable. This terminology can be extended over longer causal paths to produce ‘grandchild’ nodes and even ‘great grandchild’ nodes
Parent		A direct ancestor of a particular variable is known as a ‘parent’ of that variable. This terminology can be extended over longer causal paths to produce ‘grandparent’ nodes and even ‘great grandparent’ nodes
*Variable roles*		The role of a particular variable is defined in relation to the causal estimand of interest. These include the exposure of interest (e.g., the treatment), the outcome of interest (e.g., patient reported outcome measure), and other variables that are connected to the exposure and outcome (e.g., confounders, mediators, and colliders)
Exposure		The event or state whose causal effect is of interest (e.g., health condition or treatment). Commonly referred to by the letter ‘X’ or ‘A’, although any distinct and explicit labelling is acceptable (e.g., ‘Exposure (X)’)
Outcome		The event or state whose determination is of interest (e.g., depression or death). Commonly defined by the letter ‘Y’, although any distinct and explicit labelling is acceptable (e.g., ‘Outcome (Y)’)
Confounder		A confounder is a variable on a confounding path between the exposure and outcome, which when perfectly conditioned on would close that confounding path. This primarily includes variables that cause both the exposure and outcome, where the confounder may be known as a fork node. Commonly referred to by the letter ‘C’, ‘Z’, or ‘L’, although any distinct and explicit labelling is acceptable (e.g., ‘Confounder (C)’)
Mediator		A mediator is an intermediate event or state on a causal path between the exposure and outcome. Commonly referred to by the letter ‘M’ or ‘I’, although any distinct and explicit labelling is acceptable (e.g., ‘Mediator (M)’)
Collider		A collider is any variable that is caused by two or more other variables, the most important of which are those sitting on a non-causal path between the exposure and outcome. There is no convention for referring to colliders although the letter ‘S’ is common when the collider is related to selection into the study sample. We have used the letter ‘Z’ to reduce confusion with confounders (e.g., ‘Collider (Z)’)

*C* confounder; *DAG* directed acyclic graph; *M* mediator; *SEM* structural equation modelling; *X* exposure; *Y* outcome; *Z* collider

As described in Table 1 and shown in Fig. 1, variables (or measurements thereof) within a DAG are represented by nodes, any two of which may be connected by a unidirectional arrow (or ‘arc’) to signify that the first variable is thought to cause the second. Arcs form paths between variables which may be open (transmitting an association) or closed (not transmitting an association). Causal paths are paths where all arcs flow in the same direction, while non-causal paths are those where the arcs do not flow in the same direction. Because a DAG is acyclic, no single measure of a variable may cause itself.

The average causal effect of a specified *exposure (X)* on a specified *outcome (Y)* is the combination of all causal paths between X and Y. In theory, this may be estimated from the conditional association between X and Y, if an appropriate set of variables are conditioned so all causal paths are open and all non-causal paths are closed; formally this is known as the ‘back-door criterion’ for estimating causal effects from observed associations [17]. This requires: (1) Conditioning on *confounders* (C_1_, C_2_), which are intermediate fork nodes (C_1_) or chain nodes (C_2_) on non-causal paths between the exposure and outcome due to common causes of X and Y (i.e., due to C_1_ in X ← C_1_ → C_2_ → Y); (2) Not conditioning on *mediators* (M), which are intermediate chain nodes on causal paths between the exposure and outcome (i.e., X → M → Y); (3) Not conditioning on *colliders* (Z), which are any variables caused by two or more other variables, but are relevant when they form intermediate collider nodes on non-causal paths between the exposure and outcome (e.g., X → Z ← Y) [23, 24]. For example, in Fig. 1, to estimate the average causal effect (also known as the total causal effect) of the exposure (X) on the outcome (Y), we would need to condition on at least one of the confounders (C_1_ or C_2_) and we must not condition on the mediator (M) or collider (Z). If we wanted to estimate a direct causal effect (e.g., the part of the total causal effect that acts through X → Y but not through X → M → Y), then these can be estimated by conditioning on mediators, further details of which are described by Petersen, Sinisi, and van der Laan [25]. In the following however we focus exclusively on estimating average causal effects.

To further illustrate these concepts, consider if in Fig. 1 we are interested in the causal effect of obesity (exposure, X) on cardiovascular vascular disease (outcome, Y). Smoking is negatively correlated with body weight and a strong determinant of cardiovascular disease, so can be considered a confounder (i.e., common cause) of obesity and cardiovascular disease (e.g., smoking could be C_1_ in Fig. 1, where C_2_ would be all the ways that smoking causes cardiovascular disease that are unrelated to obesity) [26]. Obesity can increase a person’s blood pressure and cholesterol in turn increasing their cardiovascular disease risk; thus, blood pressure can be considered a mediator of obesity on cardiovascular disease (e.g., blood pressure could be M in Fig. 1) [27]. Colliders are the more complicated variable for consideration: the ‘obesity paradox’, whereby there is an association between obesity and reduced mortality (contrary to an expected increased mortality) such as in people with coronary heart disease, heart failure, and type 2 diabetes, is a famous example partly attributed (debatably) to collider stratification bias [28–30]. In Fig. 1, such bias might be introduced if we aimed to study people with heart failure, the risk of which is influenced by obesity and cardiovascular disease (e.g., heart failure could be Z in Fig. 1). By restricting the study to people with heart failure, we would be inappropriately conditioning on an intermediate collider (Z) on a non-causal path between our exposure and outcome (X → Z ← Y) and would thus introduce bias. Collider bias most commonly occurs due to non-random sampling or participation like this, or when we condition on a mediator that is also a collider (e.g., in our example, blood pressure would likely be a collider on many other unshown paths between the obesity and cardiovascular disease). For further information, we recommend the article by Tönnies et al. [30] which explains collider bias using diabetes as an example collider for the obesity-mortality causal relationship when smoking is also considered.

In addition to their popular use for identifying appropriate variables for conditioning, DAGs are also very useful aids for explaining and understanding various different forms of error and bias that are less well recognised, such as collider bias, selection bias, and other inferential biases involved in analyses of composite (e.g., multidimensional) outcome variables [31–33]. In the following, we are less focussed on the benefits of DAGs for informing appropriate variables for conditioning or for understanding specific forms of bias. Instead, we focus on the higher-level benefits of clearly communicating your assumptions about the nature of the outcome measure being analysed.

## Depicting PROMs with DAGs using reflective and formative models

Here we introduce the practice and implications of depicting unidimensional and multidimensional outcome measures within DAGs, assuming reflective and formative models. We use the unidimensional PHQ-9 and multidimensional EQ-5D as examples. Although these measures are PROMs, the logic and suggestions are relevant to any given outcome measure. Table 2 provides summarised descriptions of four key conceptual differences between reflective and formative models, based on our descriptions within this article and as inspired by a similar table by Coltman, Devinney, Midgley, and Venaik [34].

**Table 2:** Summarised descriptions of conceptual differences between reflective and formative models

	Reflective	Formative
Nature of the construct	The latent construct pre-exists independent on the measure(s) used The latent construct is probabilistically reflected by the indicators of a relevant measure	The construct is deterministically formed by the indicators within a given measure The specific construct(s) exist due to the combination of the indicators
Direction of causality	Construct *causes* the indicators. Changes in the construct is hypothesised to be reflected by changes in the indicators, i.e., there is a probabilistic relationship from the construct to the indicators	Indicators *cause* the construct. Changes in the indicators deterministically leads to changes in the construct
Characteristics of indicators	Indicators are usually highly correlated given they reflect the same underlying latent construct Dropping an indicator does not change the construct, but it may change our accuracy to capture (changes in) the latent construct	Indicators need not share a common theme/be correlated Dropping or changing a given indicator changes the specific construct
Indicator interchangeability	Indicators are interchangeable All indicators reflect the same underlying latent construct	Indicators are not interchangeable. Each indicator captures a unique component of the composite construct

This table is inspired by a similar table titled “A framework for assessing reflective and formative models: theoretical and empirical considerations” by Coltman et al. [34]

We have drawn our DAGs using Microsoft PowerPoint, but there are many free-to-use software options available, such as DAGitty: https://dagitty.net/. Other open source software for producing DAGs are described by Pitts and Fowler [35].

### Unidimensional PHQ-9

**Fig. 2:**
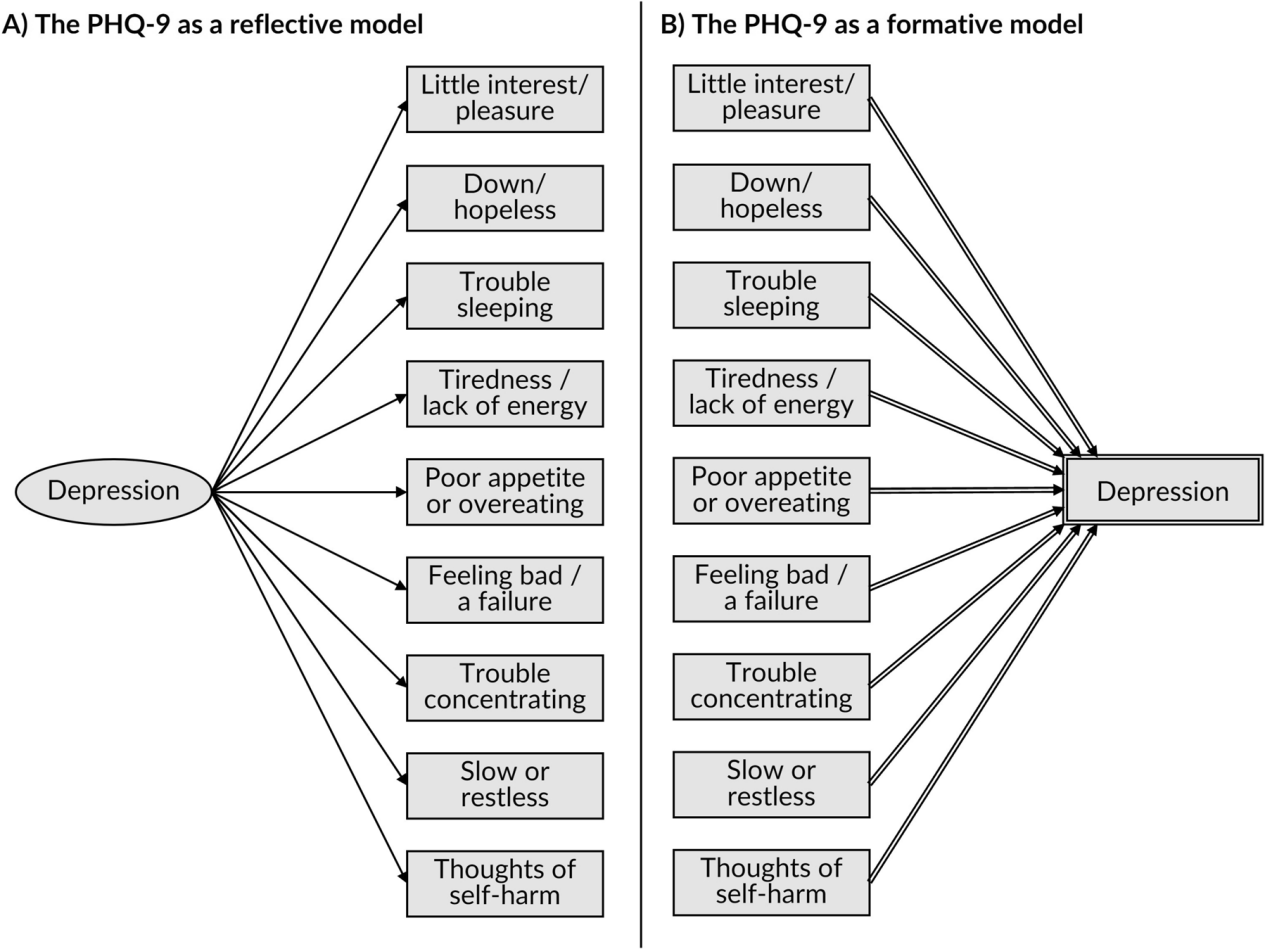
The PHQ-9 depicted within a DAG under a (a) reflective model or (b) formative model. *Footnote*: Under the reflective model (Panel **A**), depression is depicted as a latent variable by drawing the node as an ellipse; it is shown to probabilistically cause the indicators variables using ordinary arcs. Under a formative model (Panel **B**), depression is depicted as a composite derived variable by drawing the node as a double-outlined rectangle; it is shown to be mathematically determined by its parent items using double-lined arcs

Figure 2 presents the PHQ-9's indicators and construct within a DAG, under reflective and formative models. The PHQ-9 is traditionally conceptualised as reflective, i.e., the latent construct causes the indicators. However, we will examine the implications of the internal relationships being reflective or formative.

The reflective model (Fig. 2a) assumes that, although we cannot directly measure it, the construct (depression) exists in an absolute sense and manifests over time in symptoms or characteristics that are captured by the PHQ-9’s indicators (e.g., little interest/pleasure, feeling down/depressed/hopeless, tired/little energy, trouble concentrating). This is implied visually by the ordering of variables within the DAG, with an exogenous but latent depression variable occurring first and causing the symptoms later in time. In theory, any of these items, and others besides, could be used to estimate the degree of depression severity, and the exact mix of items does not alter the concept being measured, only the accuracy of our estimate.

The formative model (Fig. 2b) assumes that the concept of depression does not exist in absolute terms, but is a useful way to summarise a particular pattern of symptoms or characteristics. It assumes that a person develops a pattern and severity of symptoms, and these different patterns can be labelled as different levels of depression severity. This model makes the most sense if we conceptualise the symptoms as defining the condition, rather than being caused by it. For example, if tiredness and limited energy arise from other means (e.g., due to aging), but come together in a pattern that we call depression. Again, this is implied visually by the ordering of variables within the DAG, with the symptoms arising first and collectively determining depression. Unlike with the reflective model, the exact items that we measure and combine in a formative model determine the specific composite construct of depression that we generate. Thus, if we removed or changed some of the items in the PHQ-9, we would be measuring a different construct than the one captured by the standard PHQ-9. In Fig. 2b, this is represented using deterministic variable notation [32]. Because the depression variable is a double-outlined node, this means it is mathematically defined by its determining variables and does not exist without them.

In summary, the key difference between the reflective and formative model, while reflecting on the PHQ-9 in Fig. 2 and key terms in Tables 1 and 2, are:

i. Reflective model: depression is depicted as a latent variable for which the PHQ-9’s indictors are its probabilistic children;
ii. Formative model: depression is depicted as a composite variable which is the deterministic child to each of the PHQ-9 indictors.

While the choice between a reflective and formative model might seem rather obvious in the case of depression, both perspectives are ultimately just competing hypothesises. Presenting the hypothesized model within a DAG hence offers a simple way to demonstrate which perspective is believed to be most plausible. This becomes particularly useful when there is less certainty whether a formative or reflective model is more plausible.

### Multidimensional EQ-5D

**Fig. 3:**
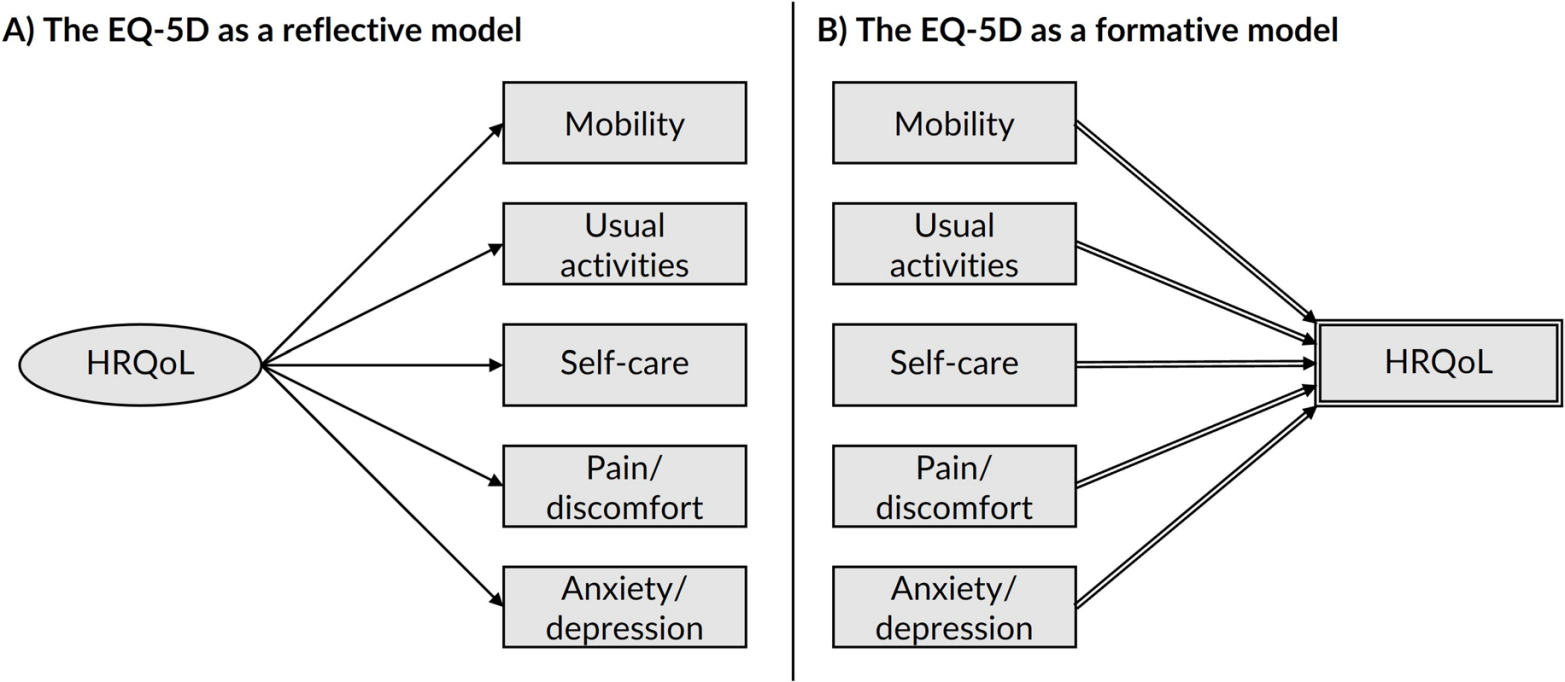
The EQ-5D depicted within a DAG under reflective model (**A**) and formative model (**B**). *Footnote*: Under the reflective model (Panel **A**), health-related quality-of-life (HRQoL) is depicted as a latent variable by drawing the node as an ellipse; it is shown to probabilistically cause the indicators variables using ordinary arcs. Under a formative model (Panel **B**), HRQoL is depicted as a composite derived variable by drawing the node as a double-outlined rectangle; it is shown to be mathematically determined by its parent items using double-lined arcs

Figure 3 presents the EQ-5D’s indicators and construct within a DAG, under reflective and formative models. Unlike the PHQ-9, the EQ-5D is designed to be multidimensional, with each item considered to be its own health-related dimension which together form a collective construct known variously as ‘generic health status’, a ‘health status profile’, or ‘health-related quality of life (HRQoL)’. Previous authors have debated whether the EQ-5D collective construct is best operationalised as reflective, formative, or a mix of the two (i.e., with some items best understood as reflective and some items best understood as formative) [36, 37]. However, such arguments are often based on data-driven rather than theoretical arguments. As with the PHQ-9, both perspectives represent competing assumptions that can be transparently shared using DAGs, preferably based on theory or mental experiment rather than compatibility with a particular dataset [38].

Figure 3a depicts the EQ-5D as a reflective model, with the collective construct, HRQoL, causing the five dimensions. This model assumes that HRQoL exists in absolute terms, regardless of how we measure it, and manifests over time through measurable variables such as mobility, self-care, usual activities, pain/discomfort, and anxiety/depression. It is assumed that HRQoL arises and causes each downstream health dimension, such that increasing HRQoL would increase the level of mobility, for example. As with the PHQ-9, this is implied visually within the DAG, with an exogenous but latent HRQoL variable occurring first and causing the other health domains later in time. In theory, we could remove or replace any of these health domains and still measure the same concept of HRQoL.

Figure 3b alternatively depicts the EQ-5D as a formative model, with the five health dimensions determining HRQoL. This model assumes that HRQoL does not exist in absolute terms, but is a useful way to summarise the other health dimensions. It is assumed that each health dimension arises and they collectively cause HRQoL, such that increasing the level of mobility would increase overall HRQoL. Again, this is implied visually within the DAG, with the health domains arising first and collectively determining HRQoL. Unlike with the reflective model, the exact items that we measure and combine determine our specific composite measure of HRQoL. Thus, if we removed mobility and self-care, for example, the resulting version of HRQoL would represent a different construct to the one captured by the standard EQ-5D. We represent this again using deterministic variable notation, with the HRQoL being a double-outlined node that is mathematically determined by the five defining variables.

In summary, the key difference between the reflective and formative model, while reflecting on the EQ-5D in Fig. 3 and key terms/descriptions in Tables 1 and 2, are:

i. Reflective model: HRQoL is depicted as a latent variable for which the EQ-5D’s indictors are its probabilistic children;
ii. Formative model: HRQoL is depicted as a composite variable which is the deterministic child to each of the EQ-5D indictors.

We believe the formative model is more plausible for HRQoL than the reflective model, in which HRQoL operates as a summary measure of health across multiple domains rather than as an independent determinant of health and functioning. As with the PHQ-9, this assumption can be clearly communicated within a DAG. However, the benefits of considering the internal relationships within a DAG extend beyond simply communicating assumptions to identifying and estimating causal effects.

### A PROM’s internal and external causal pathways: implications for causal inference

Figures 2 and 3 demonstrate how simple DAGs can be used to visually present an outcome measure’s hypothesised internal causal relationship between its indicators and construct under a reflective or formative model. Sects."Unidimensional PHQ-9" and "Multidimensional EQ-5D" similarly describe the implications for the design and interpretation of the measure; however, whether the measure is assumed to follow a reflective or formative model also has broader implications for causal inference. To explain, we consider the simple case where the outcome measure represents the causal outcome(s) of interest.

**Fig. 4:**
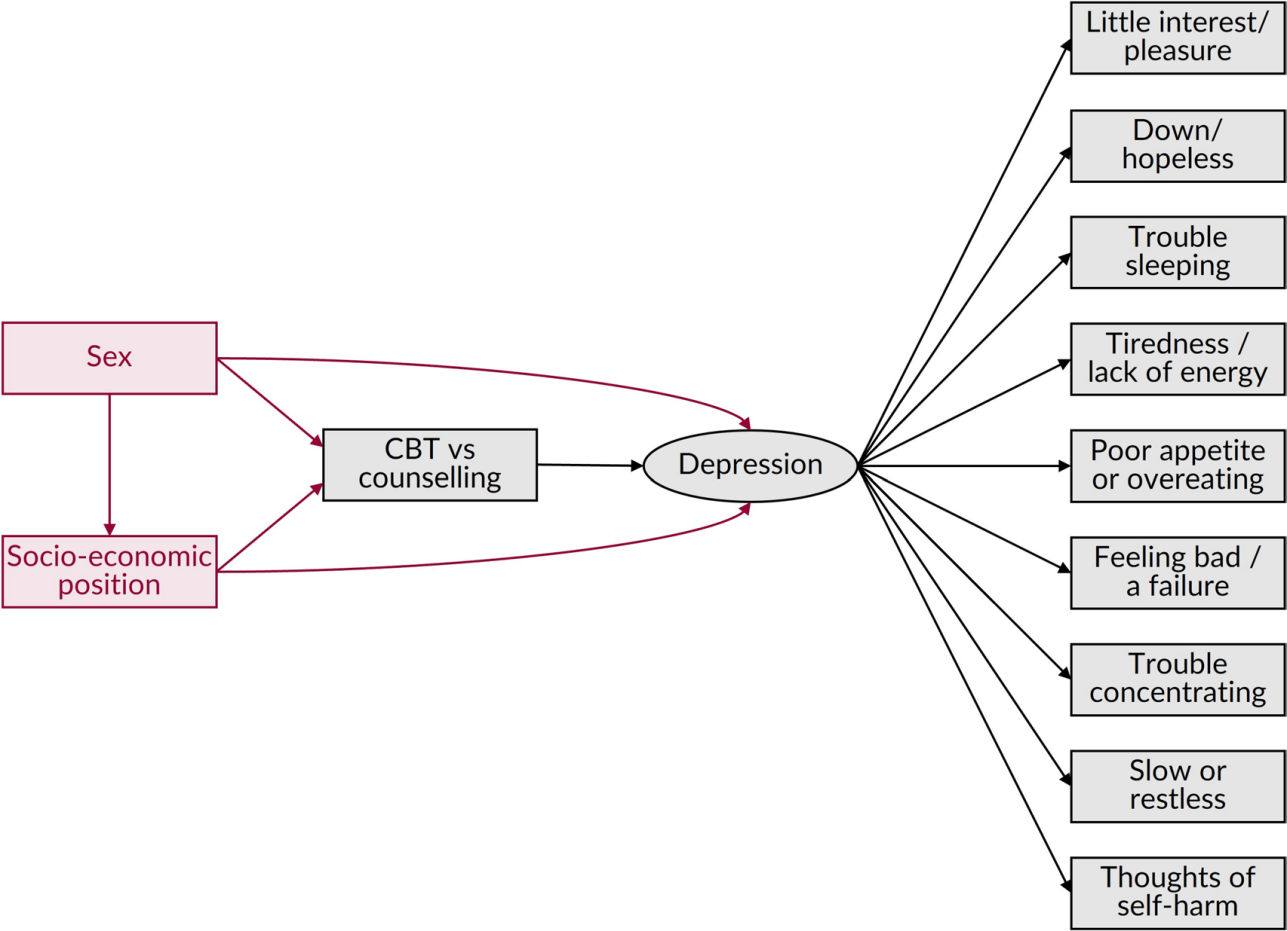
Illustrative DAG showing the relationship between CBT and depression measured using the PHQ-9 under a reflective model. *Footnote*: Under a reflective model, the items that we use to measure the outcome are assumed to be downstream of it. Although essential for measuring the outcome, in this case depression, the items are hence rather incidental to the DAG and can arguably be omitted entirely

First, let us consider a reflective model, using the example of depression severity measured by the PHQ-9. In Fig. 4, we assume the PHQ-9 is a unidimensional measure of depression. The nine indicators, which each have a Likert score from 0 (not at all) to 3 (nearly every day), thus combine to a produce a single summary score between 0 (best state) and 27 (worst state) that represents depression severity [10]. Suppose we are interested in the average causal effect of receiving CBT, instead of counselling, on depression severity using observational data. For illustrative purposes we assume sex and socio-economic position act as confounders. Since the PHQ-9 indicators are downstream of the latent depression outcome, the DAG can be drawn relatively simply, with the effect of interest represented by the direct arc between CBT and latent depression. An accurate estimate of this effect (technically the ‘latent treatment effect’ [39]) can be obtained from the association between CBT and the summary PHQ-9 score, conditioning on (e.g., by including within a regression model) sex and socio-economic position provided certain assumptions are met. These include the universal assumptions of exchangeability (no residual confounding or collider bias) [14], positivity (all levels of treatments are possible in all confounding subgroups) [40], consistency (any variations in the treatment are irrelevant to the outcome) [41], no interference (one individual’s treatment does not affect anyone else's depression) [42], and that appropriate analytical methods are used, as well as an additional assumption for latent variable outcomes known as exclusion restriction [39]. This final assumption requires that the treatment must not have any effect on the indicators other than through the latent outcome [39]. This would be violated if, for example, CBT had no effect on depression, but independently led to lower overeating, leading to a lower score on the PHQ-9 appetite item and thus a lower overall score even if the ‘true’ level of depression severity was not affected. The scope for such violations is related to the specificity of a scale’s indicators to the construct being measured, where items that represent multiple constructs offer a greater scope for violation. Many PHQ-9 items are so specific to depression (e.g., having little interest/pleasure, feeling down/hopeless, feeling bad/a failure, and having thoughts of self-harm), that it is difficult to imagine how they could change ‘independently’ of the true depression severity.

**Fig. 5:**
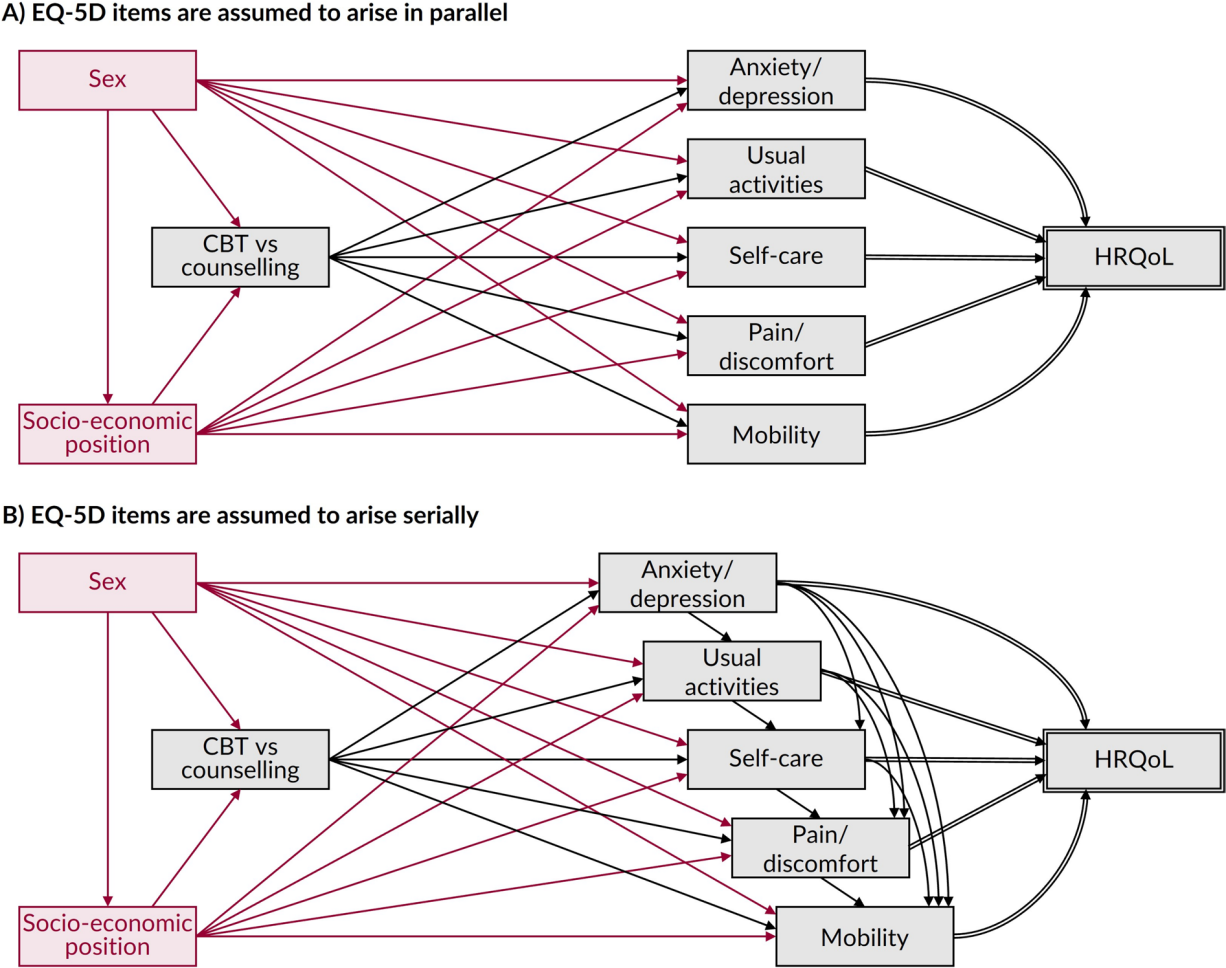
Illustrative DAGs showing the relationship between CBT and HRQoL, as measured using the EQ-5D under a formative model. Footnote: In Panel **A**, the items are assumed to occur in parallel while in Panel **B** they are assumed to occur serially, i.e., with each item arising in turn and potentially influenced by previous items. As such, in Panel **A** after controlling for the confounders (i.e., sex and socio-economic position), the remaining total causal effect on health-related quality-of-life (HRQoL) is assumed to only be attributable to ‘direct’ causal effects, i.e., directly from the exposure (cognitive behavioural therapy [CBT] or counselling) to the EQ-5D dimensions which determines the level of HRQoL. In comparison, in Panel **B** after controlling for the confounders, the remaining total causal effect on HRQoL is assumed to be a combination of ‘direct’ causal effects (i.e., directly from the exposure) and ‘indirect’ causal effects (i.e., mediated via specific EQ-5D domains having a causal effect on another EQ-5D domain, e.g., anxiety/depression→ usual activities→ self-care→ pain/discomfort→ mobility). Depending on your causal effect of interest (e.g., total or only direct causal effects), Panel **A** may represent a simpler variable relationship network than Panel **B** for causal analysis

Second, let us consider a formative model, using the example of HRQoL measured by the EQ-5D. In Fig. 5, we assume the EQ-5D is a multidimensional measure of HRQoL, but the final form of the DAG depends on whether we believe the items arise in parallel (Fig. 5a) or serially in time (Fig. 5b). Regardless, the five dimensions are rated on 3-point or 5-point scale (depending on the EQ-5D version) which generates a health state profile score (e.g., 12321); subsequently one of the more common ways to use the EQ-5D is that a utility-based (aka. preference-based) value set is assigned, with each health state profile being assigned a utility value which is considered to represent HRQoL [2, 13]. Suppose we were interested in the average causal effect of CBT, instead of counselling, on HRQoL using routine data. Again, we assume sex and socio-economic position act as confounders. Since the EQ-5D indicators are upstream of the composite outcome, the DAG is now more difficult to draw, since the effect of interest is a combination of the causal effects on all five dimensions. This brings a few well-known limitations, not least the potential to obscure potentially important effects for particular components [43]. The identifiability assumptions must now also be met for all the constituent dimensions, meaning that, in our example, all sources of confounding and collider bias must now be identified and closed for all five dimensions. If the items arise serially in time (Fig. 5b), then the DAG becomes more difficult to draw and the effect potentially more difficult to identify because the exposure will need to clearly precede all components of the outcome to avoid reverse causality bias (e.g., instead of X causing Y, Y causes X).

Inherently, the ability to estimate causal effects relies on various assumptions and conditions to be met; however, the main intention of this section is to show that key aspects for consideration (i.e., hypothesised causal and non-causal pathways) can be drawn visually using DAGs, then appropriate structural or analytical methods chosen to support estimating such causal effects, which has specific considerations when the outcome is represented by an outcome measure like a PROM.

## Discussion

PROMs are an essential way to measure important health-related outcomes and are thus of huge interest to health researchers and practitioners. Even where a PROM is not intended for causal inference, understanding the internal causal relationship between the items and intended construct(s) of interest can have important implications for both the measure’s development and use. However, in practice, many routine health and healthcare research questions are likely to be causal and hence would benefit from using methods that have been explicitly developed for considering and estimating causal effect [44].

In this article, we have provided a brief introduction to causal DAGs as a popular aid to estimating causal effects in observational data, but also a visually compelling means to identify and communicate assumptions about the nature of a PROM. We also explored the philosophical differences between PROMs that are assumed to follow reflective and formative models, then considered the implications for identifying and estimating causal effects. As such, we have also visually shown that multidimensional outcome constructs formed under a formative model increases the complexity of causal analyses, which may seem off-putting for some researchers interested in causal inference. Despite this complexity, multidimensional measures are widely used, such as for clinical outcome assessment, and may aid with a range of outcome studies, such as ‘outcome-wide’ epidemiology when assessing if an exposure may be beneficial for some outcomes but harmful for others [45, 46].

Outcome-wide epidemiology has become popular and may be benefited by using PROMs, whereby you seek to assess the causal effect of a single exposure on multiple outcomes [45]. VanderWeele [45] stressed the public health importance of outcome-wide epidemiology, despite challenges in confounding control which is related to the challenges we presented for estimating causal effects on a multidimensional outcome construct formed under a formative model. Outside epidemiology, outcome-wide studies are, in a more general sense, relevant to a range of observational settings (i.e., outside the context of RCTs) where PROMs could have a role as a causal outcome of interest; for example related to non-trial-based clinical outcome assessment, routine care monitoring, health service evaluation, health technology assessment, outcomes-based commissioning, value-based pricing, health economics and outcomes research, and more generalised population/public health research [46–51]. Thus, our article may also aid with bridging a gap between the disciplines of epidemiology and other observational research across a range of disciplines, within which causal inference and PROMs are potentially of key importance.

## Conclusion

Many studies using PROMs have causal aims, even if these are not explicit. Using causal inference methods, including DAGs, will hopefully help to make these aims more explicit. The use of DAGs can aid in any study when there is an interest in the effect of an exposure on outcome(s) which may be represented by a PROM. By explicitly depicting PROMs within DAGs, these methods promise to help PROM users to think more deeply about the nature of their measure, and the interpretation of analyses that rely on these instruments.

## Data Availability

Not applicable as the manuscript uses no human or animal subject data.

## Code availability

Not applicable as the manuscript involves no code for analysis.
